# *In Vivo* Efficacy of Umbilical Cord Blood Stem Cell-Derived NK Cells in the Treatment of Metastatic Colorectal Cancer

**DOI:** 10.3389/fimmu.2017.00087

**Published:** 2017-02-06

**Authors:** John P. Veluchamy, Silvia Lopez-Lastra, Jan Spanholtz, Fenna Bohme, Nina Kok, Daniëlle A. M. Heideman, Henk M. W. Verheul, James P. Di Santo, Tanja D. de Gruijl, Hans J. van der Vliet

**Affiliations:** ^1^Department of Medical Oncology, VU University Medical Center, Cancer Center Amsterdam, Amsterdam, Netherlands; ^2^Glycostem Therapeutics, Oss, Netherlands; ^3^Innate Immunity Unit, Institut Pasteur, Paris, France; ^4^Institut National de la Santé et de la Recherche Médicale (INSERM) U1223, Paris, France; ^5^Université Paris-Sud (Paris-Saclay), Paris, France; ^6^Department of Pathology, VU University Medical Center, Amsterdam, Netherlands

**Keywords:** EGFR, RAS mutation, cetuximab, metastatic colorectal cancer, A-PBNK, UCB-NK, allogeneic NK cell immunotherapy

## Abstract

Therapeutic monoclonal antibodies against the epidermal growth factor receptor (EGFR) act by inhibiting EGFR downstream signaling and by eliciting a natural killer (NK) cell-mediated antitumor response. The IgG_1_ mAb cetuximab has been used for treatment of RAS^wt^ metastatic colorectal cancer (mCRC) patients, showing limited efficacy. In the present study, we address the potential of adoptive NK cell therapy to overcome these limitations investigating two allogeneic NK cell products, i.e., allogeneic activated peripheral blood NK cells (A-PBNK) and umbilical cord blood stem cell-derived NK cells (UCB-NK). While cetuximab monotherapy was not effective against EGFR^−^ RAS^wt^, EGFR^+^ RAS^mut^, and EGFR^+^ BRAF^mut^ cells, A-PBNK were able to initiate lysis of EGFR^+^ colon cancer cells irrespective of RAS or BRAF status. Cytotoxic effects of A-PBNK (but not UCB-NK) were further potentiated significantly by coating EGFR^+^ colon cancer cells with cetuximab. Of note, a significantly higher cytotoxicity was induced by UCB-NK in EGFR^−^RAS^wt^ (42 ± 8 versus 67 ± 7%), EGFR^+^ RAS^mut^ (20 ± 2 versus 37 ± 6%), and EGFR^+^ BRAF^mut^ (23 ± 3 versus 43 ± 7%) colon cancer cells compared to A-PBNK and equaled the cytotoxic efficacy of the combination of A-PBNK and cetuximab. The antitumor efficacy of UCB-NK cells against cetuximab-resistant human EGFR^+^ RAS^mut^ colon cancer cells was further confirmed in an *in vivo* preclinical mouse model where UCB-NK showed enhanced antitumor cytotoxicity against colon cancer independent of EGFR and RAS status. As UCB-NK have been proven safe in a recently conducted phase I clinical trial in acute myeloid leukemia, a fast translation into clinical proof of concept for mCRC could be considered.

## Introduction

Colorectal cancer (CRC) is the fourth leading cause of cancer-related deaths in the world (1). Despite substantial advances in the treatment of metastatic CRC (mCRC) over the last decades that have contributed to better survival rates (2, 3), the disease is still frequently fatal. Monoclonal antibodies (mAbs) targeting the epidermal growth factor receptor (EGFR) pathway, such as panitumumab and cetuximab, are approved for the treatment of patients with advanced CRC either in combination with chemotherapy or, as monotherapy, in chemorefractory conditions (4). Cetuximab (CET) and panitumumab block the interaction between EGFR and its ligands, thus inhibiting the downstream RAS-signaling cascade and tyrosine kinase activation (5). However, mutations in tumor suppressor genes and proto-oncogenes in EGFR signaling pathways, such as in RAS, BRAF, and PIK3CA, are common in patients with CRC. These mutations represent a poor prognostic marker and render anti-EGFR mAbs ineffective, leaving 42% of the chemorefractory mCRC population without this standard treatment option (6, 7).

Besides the blockade of the EGFR–ligand interaction on tumor cells, therapeutic mAbs can also interact with natural killer (NK) cells triggering antibody-dependent cell-mediated cytotoxicity (ADCC) (8–10), and this can translate into superior antitumor effects (11). Two NK cell subsets can be identified based on the expression of CD16, the low affinity FcγRIIIa receptor. The majority of NK cells are CD56^dim^CD16^+^ and play an active role in NK cell cytotoxicity and are capable of performing ADCC upon IgG_1_ engagement via CD16, whereas CD56^bright^CD16^−^ NK cells are mainly immune regulatory in function, secreting cytokines, and are less cytotoxic than CD56^dim^ cells (12). NK cell functions are tightly regulated by a delicate balance between activating receptors (like the natural cytotoxicity receptors NKp46, NKp30, and NKp44, or C-type lectin-like receptor NKG2D) (13) and major histocompatibility complex (MHC) class I binding inhibitory receptors, including killer cell immunoglobulin-like receptors (KIRs), LIR1/ILT2, and NKG2A/CD94 (14). The importance of NK cells in controlling tumors has been extensively demonstrated since their identification 40 years ago (15–17).

Several studies have shown a dysfunctional phenotype and poor infiltration of NK cells in the CRC tissue from early stages on, together with an immunosuppressive tumor microenvironment (18, 19). Hence, various strategies, e.g., using cytokines or therapeutic ADCC enhancing mAbs, have been explored to increase NK cell numbers and function and to enhance their trafficking to tumor sites (20). Another approach entails the adoptive transfer of *in vitro* manipulated and expanded autologous or allogeneic NK cells. Autologous NK cells so far have failed to demonstrate significant therapeutic benefits in solid tumors (21–23). Therefore, the focus has shifted to the development of allogeneic NK cells as a potential adoptive cell therapy for treatment in solid tumors. Previously, we demonstrated that the combination of allogeneic activated peripheral blood NK cells (A-PBNK) and CET can effectively target RAS mutant (RAS^mut^) CRC tumors (24).

Here, we compared two feeder cell-free allogeneic NK cell products, i.e., A-PBNK and umbilical cord blood stem-cell derived NK cells (UCB-NK), alone or in combination with cetuximab for antitumor effects against RAS^mut^ CRC.

## Materials and Methods

### Cell Lines

Cell lines A431 (epidermoid carcinoma), COLO320, SW480, and HT-29 (colon carcinoma) were obtained from American Type Culture Collection and cultured in Dulbecco’s modified medium (DMEM; Invitrogen, Carlsbad, CA, USA) containing 100 U/ml penicillin, 100 µg/ml streptomycin, and 10% fetal calf serum (FCS; Integro, Zaandam, The Netherlands). Cell cultures were passaged every 5 days and maintained in a 37°C, 95% humidity, 5% CO_2_ incubator.

### PBNK Isolation and Activation

Peripheral blood mononuclear cells (PBMCs) were isolated from the heparinized blood of healthy donors (six males, four females, age range = 56–64 years and CRC patients (eight males, two females, age range = 66–74 years) after written informed consent and according to protocols approved by the institutional review board of VU University Medical Center, Amsterdam (NCT01792934). Blood samples were collected at baseline and after the first cycle of first-line palliative chemotherapy consisting of oral capecitabine (1,000 mg/m^2^, bid, days 1–14), i.v. oxaliplatin (130 mg/m^2^, day 1), and i.v. bevacizumab (7.5 mg/kg, day 1, in 4/10 mCRC patients). PBMCs were isolated using Lymphoprep™ (STEMCELL Technologies, Cologne, Germany) density gradient centrifugation. CD56^+^ NK cells were isolated from PBMC using a MACS Human NK cell isolation kit (Miltenyi Biotec, Bergisch Gladbach, Germany) according to the manufacturer’s instructions. PBNK cells purity and viability were checked using CD3 VioBlue, CD56 APC Vio 770, and CD16 APC (Miltenyi Biotech) and 7-AAD (Sigma Aldrich, Zwijndrecht, The Netherlands). Isolated PBNK cells were activated overnight with 1,000 U/ml IL-2 (Proleukin^®^; Chiron, München, Germany) and 10 ng/ml IL-15 (CellGenix) for use in cytotoxicity assays. The parameters compared before and after stimulation with cytokines were NK purity (87 ± 5 versus 84 ± 2%), NK CD16^+^, (92 ± 12 versus 88 ± 8%) and NK viability (89 ± 5 versus 84 ± 8%), respectively.

### Flow Cytometry

The antibody staining mix for the assessment of NK cell functionality consisted of CD45 VioGreen, CD14 VioBlue, CD19 VioBlue, and SYTOX^®^ Blue, together with CD3 PerCP-Vio 700 and TCRγδ PerCP-Vio700 to exclude dead cells, debris, and non-NK populations from PBMCs. NK cells were identified by the expression of CD45^+^CD3^−^CD56^+^ cells, and further characterized for NK functionality by plotting against CD16 APC, CD25 VioBrightFITC, CD107a PE, and NKp44 PE-Vio770 and for NK cell phenotype by plotting against NKG2A PE-Vio770, NKG2C PE, NKG2D PerCP-Cy5.5, and PanKIR2D FITC. All antibodies were supplied by Miltenyi Biotec except SYTOX^®^ Blue (Thermo Fisher Scientific, Berlin, Germany).

### UCB-NK Cultures

Allogeneic NK cells (UCB-NK) were generated from cryopreserved umbilical cord blood (UCB) hematopoietic stem cells as previously described (25). CD34^+^ UCB cells from six UCB-donors were plated (4 × 10^5^/ml) into 12-well tissue culture plates (Corning Incorporated, Corning, New York, NY, USA) in Glycostem Basal Growth Medium (GBGM^®^) (Clear Cell Technologies, Beernem, Belgium) supplemented with 10% human serum (HS; Sanquin Bloodbank, Amsterdam, The Netherlands), 25 ng/ml of SCF, Flt-3L, TPO, and IL-7 (CellGenix, Freiburg, Germany). In the expansion phase II, from day 9 to day 14, TPO was replaced with 20 ng/ml IL-15 (CellGenix). During the first 14 days of culture, low molecular weight heparin (Clivarin^®^; Abbott, Wiesbaden, Germany) in a final concentration of 20 µg/ml and a low-dose cytokine cocktail consisting of 10 pg/ml GM-CSF (Neupogen), 250 pg/ml G-CSF and 50 pg/ml IL-6 (CellGenix) were added to the expansion cultures. Cells were refreshed with new medium twice a week and maintained at 37°C, 5% CO_2_. On day 14, the NK cell differentiation process was initiated by addition of NK cell differentiation medium consisting of the same basal medium with 2% HS but with high-dose cytokine cocktail consisting of 20 ng/ml of IL-7, SCF, IL-15 (CellGenix), and 1,000 U/ml IL-2 (Proleukin^®^; Chiron, München, Germany). Cultures were refreshed every 2–3 days and maintained till day 42. Five UCB-NK cultures were used for cytotoxicity assays and one UCB-NK culture for *in vivo* studies (both with a CD56^+^ cell purity of >95%). UCB-NK CD16 levels in matured UCB-NK cells were monitored using an antibody mix of human CD45VioGreen (1:11), CD56 APC-Vio770 (1:11), and CD16 APC (1:11). Similarly, UCB-NK CD16 expression in BRGS mice was monitored using an antibody mix of BV650 anti-mouse CD45 (clone 30-F11), Alexa Fluor^®^ 700 anti-human CD45 (clone HI30), PE-CF594 anti-human CD56 (clone B159), all from BD, and APC-Vio770 anti-human CD56 (clone REA196) and APC CD16 (clone REA423) both from Miltenyi Biotec.

### NK Cell Cytotoxicity Assays

Flow cytometry was used for the readout of cytotoxicity assays. Target cells (COLO320, SW480, and HT-29) were labeled with 5 µM pacific blue succinimidyl ester (PBSE; Molecular Probes Europe, Leiden, The Netherlands) at a concentration of 1 × 10^7^ cells/ml for 10 min at 37°C. The reaction was terminated by adding an equal volume of FCS, followed by incubation at room temperature for 5 min after which stained cells were washed twice and suspended in DMEM + 10% FCS to a final concentration of 5 × 10^5^/ml. Overnight activated PBNK cells and UCB-NK cells were washed with PBS and suspended in GBGM + 2% FCS to a final concentration of 5 × 10^5^/ml. Target cells were cocultured with effector cells at an E:T ratio of 1:1 in a total volume of 250 µl in 96-well flat-bottom plates (5 × 10^4^ targets in 100 µl of DMEM + 10% FCS incubated with 5 × 10^4^ effectors in 100 µl of GBGM + 2% FCS, further supplemented with 25 µl of GBGM + 2% FCS and DMEM + 10% FCS medium). NK cells and target cells alone were plated out in triplicate as negative controls. Target cells were coated with 5 µg/ml cetuximab (Merck, Darmstadt, Germany) for 1 h at 4°C. To measure degranulation of NK cells, anti-CD107a PE (Miltenyi Biotech) was added in 1:20 dilution at the beginning of the assay. After incubation for 4 h at 37°C, cells were harvested and stained with CD56 APC Vio 770 (1:25) and CD16 APC (1:25) (Miltenyi Biotech) and 7-AAD (1:500) (Sigma Aldrich). Degranulation of NK cells was measured by detecting cell surface expression of CD107a.

### *In Vivo* Studies

The EGFR^+^RAS^mut^ SW480 cell line and EGFR^+++^RAS^wt^ A431 cell line were stably transduced with Gaussia Luciferase (Gluc) for *in vivo* studies. Lentiviral (LV) supernatant of Cerulean Fluorescent Protein (CFP)-positive Gluc virus (LV-CFP-Gluc) was kindly provided by Dr. Tom Würdinger (26). SW480 and A431 cells with Gluc expression of 95% were used for mouse studies.

Immunodeficient BRGS mice (BALB/c *Rag2*^tm1Fwa^ *Il2rg*^tm1Cgn^ *Sirpa^NOD^*) were used in this study. Twenty-four adult mice (male, 8 weeks old) received an intravenous (i.v.) tail vein injection with 0.5 × 10^6^ SW480 Gluc cells at day 0 and were randomized into four groups. Group A only received SW480 cells, group B received SW480 in combination with cetuximab intraperitoneally (i.p., 0.5 mg, days 1, 4, and 7), group C received SW480 in combination with UCB-NK i.v. (1 × 10^7^, days 1, 4, and 7), and group D received SW480 cells in combination with UCB-NK i.v. (1 × 10^7^, days 1, 4, and 7) and cetuximab i.p. (0.5 mg, days 1, 4, and 7). Groups C and D received i.p. 0.5 µg IL-15 + 7.5 µg IL-15Rα every 2–3 days from day 0 till day 14. Further, three adult mice received i.v. tail vein injection of 0.5 × 10^6^ A431 Gluc cells at day 0 and were treated with 0.5 mg cetuximab (i.p., 0.5 mg, days 1, 4, and 7), which were used as a cetuximab efficacy control. Treatment effects were monitored using blood Gluc levels and bioluminescence imaging (BLI). All manipulations of BRGS mice were performed under laminar flow conditions.

### Blood Gluc Quantification *In Vitro*

Secreted Gluc was measured according to a protocol described previously (27). A total of 10 µl of blood was collected by capillarity into EDTA containing Microvette^®^ CB tubes. Blood samples were distributed in 96-well black plates then mixed with 100 µl of 100 mM Gluc substrate native coelenterazine in PBS (P.J.K. GmbH, Kleinblittersdorf, Germany), and 5 min later, light emission was quantified. Blood that was withdrawn before tumor inoculation served to determine a baseline value. Measurements were done twice a week until day 35. Gluc activity was measured using IVIS spectrum luminescence detector (PerkinElmer, Villebon-sur-Yvette, France). Data obtained were quantified using Living Image 4.0 software (PerkinElmer, Villebon-sur-Yvette, France).

### BLI *In Vivo*

Mice were anesthetized using isofluorane gas in an induction chamber at a gas flow of 2.5 pm. Retro-orbital injection of coelenterazine (4 mg/kg body weight) was administered and mice were placed in the anesthesia manifold inside the imaging chamber and imaged within 5 min following substrate injection. Mice were placed into the light chamber and overlay images were collected for a period of 15 min using IVIS spectrum *in vivo* imaging system (PerkinElmer, Villebon-sur-Yvette, France). Images were then analyzed using Living Image 4.0 software (PerkinElmer, Villebon-sur-Yvette, France).

### Ethics Statement

Animals were housed in isolators under pathogen-free conditions with humane care and anesthesia was performed using inhalational isoflurane anesthesia to minimize suffering. Experiments were approved by the Institut Pasteur’s ethical committee for animal use in research, Comité d’étique en expérimentation animale (CETEA) #89, protocol reference # 2007–006 and validated by the French Ministry of Education and Research (Reference # 02162.01).

### Statistical Analysis

Data were analyzed using GraphPad Prism version 6 (GraphPad Software, San Diego, CA). Differences between conditions were determined using one-way ANOVA or two-way ANOVA with multiple comparisons between column means, unpaired *t* test and log-rank (Mantel–Cox) test as deemed appropriate. A *P*-value of <0.05 was considered statistically significant.

## Results

### Highly Dysfunctional NK Cells in CRC Patients

**Figure 1:**
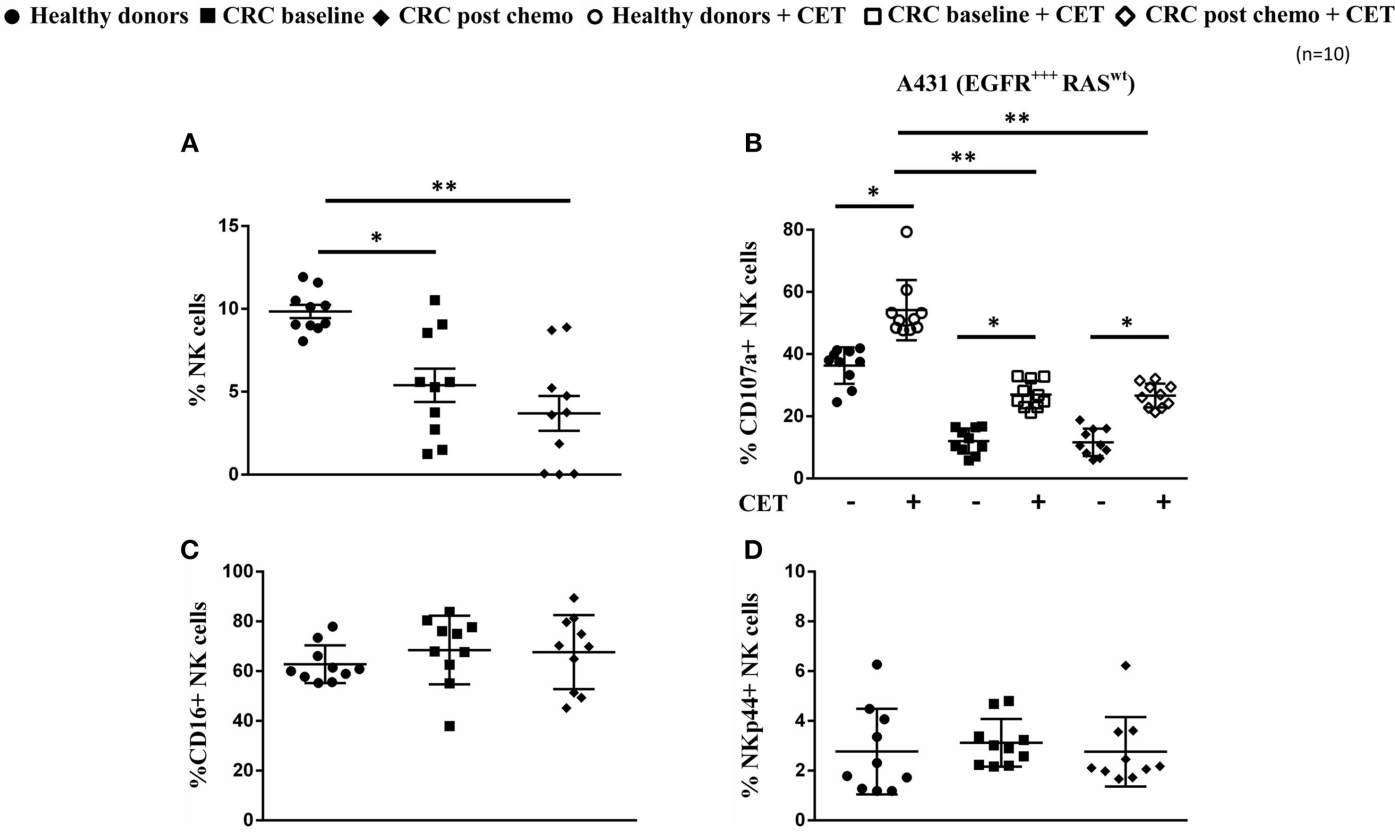
**Low prevalence and functionally impaired natural killer (NK) cells in colorectal cancer (CRC) patients**. **(A)** Frequency of NK cells within peripheral blood mononuclear cells from healthy controls and from metastatic CRC (mCRC) patients at baseline and after the first cycle of chemotherapy. **(B)** NK cell degranulation in healthy controls and mCRC patients after a 4-h coculture of resting NK cells with A431 cells in the presence (open symbols) or absence (closed symbols) of cetuximab at an E:T ratio of 1:1. **(C)** Expression levels of resting NK cell CD16 and **(D)** NKp44 in healthy controls and in mCRC patients before and after one cycle of chemotherapy. Data represent mean ± SEM from 10 mCRC patients and 10 age- and sex-matched healthy controls. *P < 0.05, **P < 0.01, ***P < 0.005, calculated with one-way ANOVA, multiple comparison between column means.

Flow cytometry was used to determine the frequency, phenotype, and functionality of NK cells in PBMC of healthy volunteers (*n* = 10, age range 56–64 years, 6 males/4 females) and patients with metastatic CRC (*n* = 10, age range 66–74 years, 8 males/2 females) before and after the first cycle of first-line palliative chemotherapy consisting of oral capecitabine (1,000 mg/m^2^, bid, days 1–14), i.v. oxaliplatin (130 mg/m^2^, day 1), and i.v. bevacizumab (7.5 mg/kg, day 1, in 4/10 mCRC patients). As illustrated in Figure 1A, mCRC patients harbored on average a 20% lower percentage of CD3^−^CD56^+^NK cells in the total CD45^+^ lymphocyte population as compared to healthy controls (*P* < 0.05). These lower NK rates, which are in line with a previous report in CRC (28), further declined after the first cycle of chemotherapy (*P* < 0.01).

We next evaluated whether this quantitative NK cell defect was also accompanied by functional defects in the NK cell population. For this purpose, the ability of NK cells from healthy volunteers and mCRC patients to induce both natural cytotoxicity and mediate ADCC of the epidermoid carcinoma cell line A431 (MHC-I^low^, EGFR^high^, KRAS^wt^) was assessed. For ADCC, tumor target cells were coated with cetuximab before the addition of NK cells. It was evident that the cytotoxic potential of NK cells from mCRC patients, as reflected by degranulation (i.e., CD107a surface expression), was highly impaired both before chemotherapy and after the first cycle of chemotherapy. Though NK cells of mCRC patients were capable of ADCC, as evidenced by significant increases in degranulation when target cells were coated with cetuximab (*P* < 0.05), levels were still low compared to those observed in healthy volunteers (Figure 1B). Of note, although the NK cells of healthy volunteers and mCRC patients expressed similar levels of CD16 (Figure 1C), this did not translate into comparable levels of ADCC. NKp44 expression, known to reflect the activation status of NK cells, was similar between the HD and mCRC groups used in NK cytotoxicity experiments (Figure 1D). Furthermore, no significant differences were observed in expression levels of NK activating (NKG2D, NKG2C) and NK inhibiting (NKG2A, KIR2D) receptors between healthy controls and CRC patients (Figure S1 in Supplementary Material).

### Enhanced *In Vitro* Cytotoxicity of Colon Cancer Cells Mediated by UCB-NK Cells

**Figure 2:**
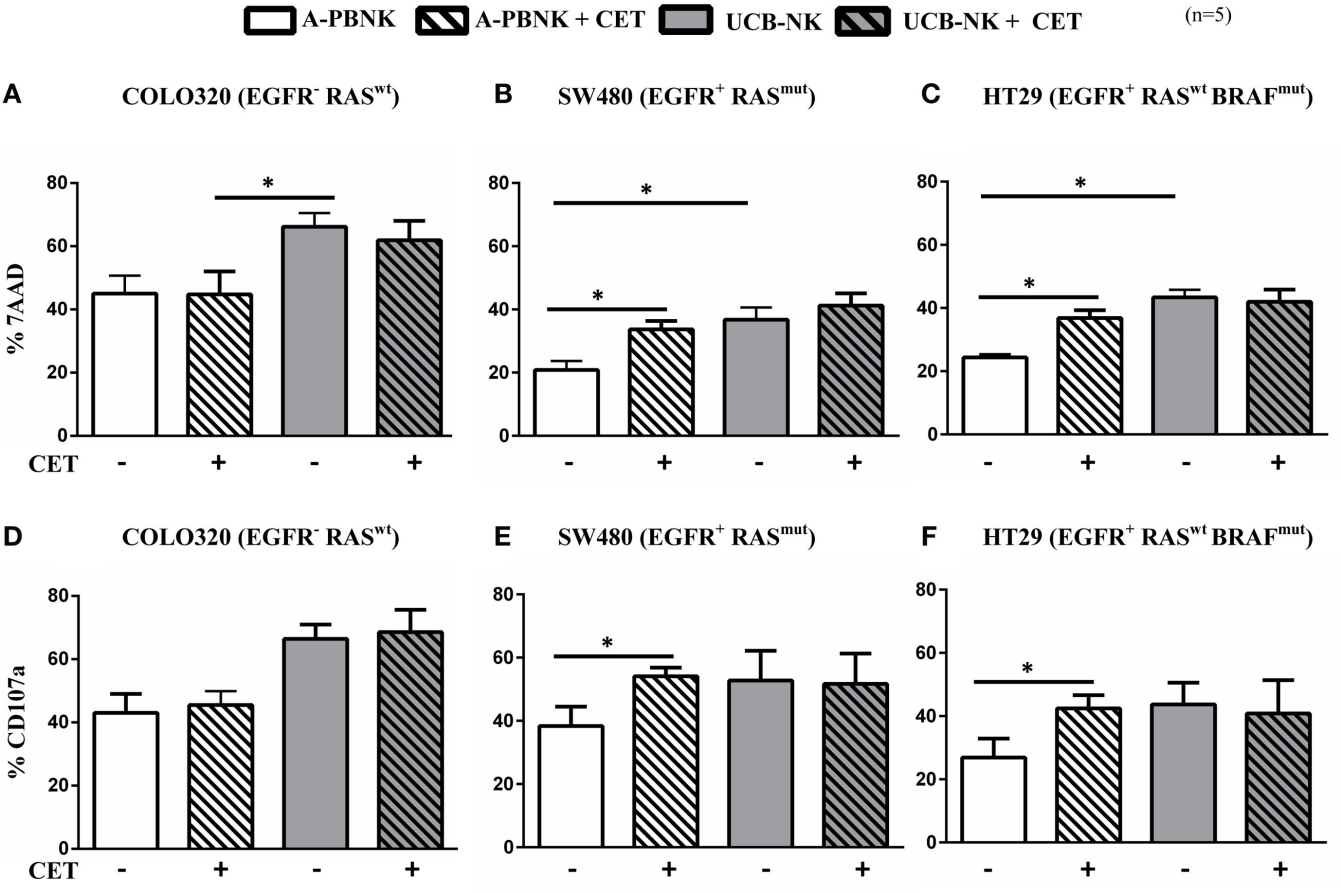
***Ex vivo* cytotoxic efficacy of A-PBNK and UCB-NK cells against colorectal cancer (CRC) cells**. CRC cell lines COLO320 (EGFR^−^, RAS^wt^), SW480 (EGFR^+^, RAS^mut^), and HT-29 (EGFR^+^, RAS^wt^, BRAF^mut^) were subjected to natural killer (NK) killing using two allogeneic NK cell products, i.e., A-PBNK and UCB-NK cells. 7-AAD **(A,B,C)** and CD107a **(D,E,F)** were measured after a 4-h coculture of A-PBNK and UCB-NK cells with CRC targets in the presence or absence of cetuximab at an E:T ratio of 1:1. Experiments were carried out in triplicate. Bars represent mean ± SEM, *n* = 5. *P < 0.05 and **P < 0.01, calculated with two-way ANOVA, multiple comparison between column means.

In order to explore novel therapies to replace dysfunctional NK cells in patients with advanced CRC, we tested two different sources of allogeneic NK cell products (A-PBNK and UCB-NK) that could eventually be used for adoptive transfer strategies. We next compared the activity of A-PBNK cells (age range 22–37 years) and UCB-NK cells using a flow based NK cell cytotoxicity assay based on detection of 7-AAD accumulation in tumor cells. Three different cell lines of colon cancer origin were compared, i.e., COLO320 (EGFR^−^ RAS^wt^), SW480 (EGFR^+^ RAS^mut^), and HT-29 (EGFR^+^ RAS^wt^ BRAF^mut^). As expected, addition of cetuximab to EGFR^−^ RAS^wt^ COLO320 cells did not result in increased killing. Of interest, lysis was consistently and significantly higher (*P* < 0.01) using UCB-NK compared to A-PBNK. As reported previously, the combination of cetuximab and A-PBNK resulted in increased killing of EGFR^+^RAS^mut^ SW480 and EGFR^+^ BRAF^mut^ HT-29 via ADCC (24). CD16 was expressed by 88 ± 8% (*n* = 5) of A-PBNK after overnight stimulation with cytokines and by 7 ± 2% (*n* = 5) of UCB-NK cells at the end of the 35-day culture period. No added effect of cetuximab was observed when using UCB-NK cells, which is possibly related to their lower *in vitro* CD16 levels (29). Of note, tumor cell lysis induced by UCB-NK cells was comparable to that observed with the combination of A-PBNK and cetuximab (Figures 2A,B,C). Measurements of NK cell degranulation reflected equivalent trends observed for tumor cell lysis (Figures 2D,E,F). These results show that UCB-NK cells have superior cytotoxic efficacy over A-PBNK cells against cetuximab-resistant colon cancer cells *in vitro*.

### UCB-NK Cells Inhibit *In Vivo* Tumor Growth and Increase Survival

**Figure 3:**
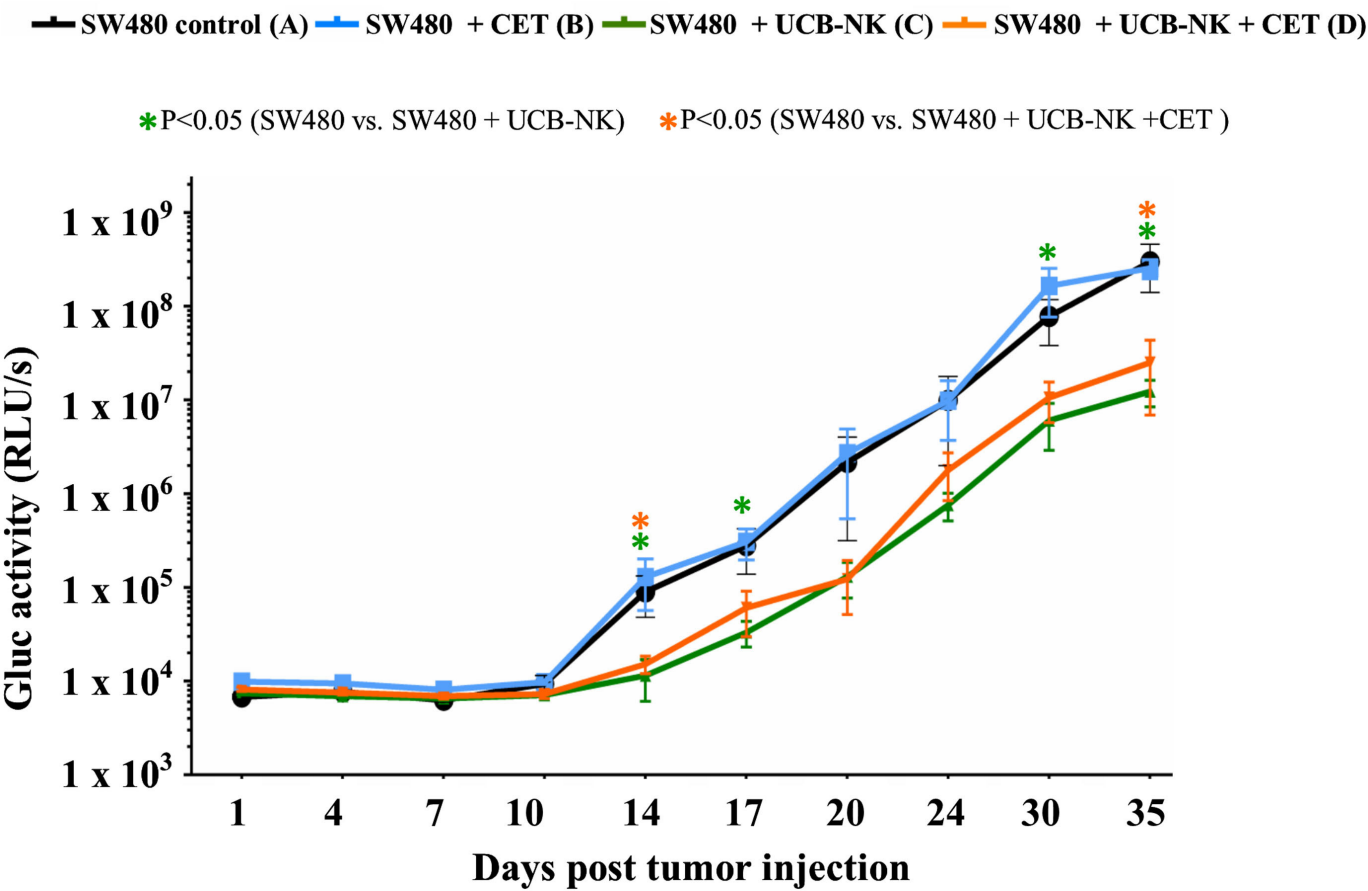
**Significant antitumor effects of UCB-NK cells *in vivo***. Real-time monitoring of tumor progression and treatment response was performed measuring Gluc levels from mice blood twice a week. Baseline Gluc values were obtained from all mice a day before tumor injection (day 1), and further monitoring continued until day 35. Blood Gluc levels were compared between control SW480 only (A) group and treatment groups SW480 + cetuximab (B), SW480 + UCB-NK (C), and SW480 + UCB-NK + cetuximab (D) for statistical significance. Data presented is from six mice per group (*n* = 6). Scatter plots represent mean ± SEM. *P < 0.05, calculated with unpaired *t* test.

To address whether UCB-NK cells exhibit similar antitumor efficacy *in vivo*, we transferred Gluc transduced SW480 cells to immunodeficient mice (BRGS; see Materials and Methods). SW480 cells are EGFR^+^RAS^mut^ and cetuximab monotherapy resistant. Mice were divided into four groups of six mice per group: SW480 only (group A), SW480 + cetuximab (group B), SW480 + UCB-NK (group C), and SW480 + UCB-NK + cetuximab (group D). Gaussia luciferase activity in whole blood was measured every 3 days to monitor the tumor burden (Figure S2 in Supplementary Material). These data confirmed our *in vitro* observations that SW480 cells were resistant to cetuximab-mediated growth inhibition (blue line). Of note, while treatment with UCB-NK cells alone significantly decreased the tumor load (green line), this effect was not increased by combining UCB-NK cells with cetuximab and thereby further confirmed both the inefficacy of cetuximab in treating RAS mutated tumors as well as the inability of cetuximab to induce ADCC of UCB-NK cells *in vivo* (orange line) (Figure 3). CD16 expression levels on UCB-NK cells were monitored in two mice upon adoptive transfer and increased from 6.0% before transfer to 14.0% (mouse 1) and 19.1% (mouse 2) at day 5 post UCB-NK cell infusion (data not shown).

**Figure 4:**
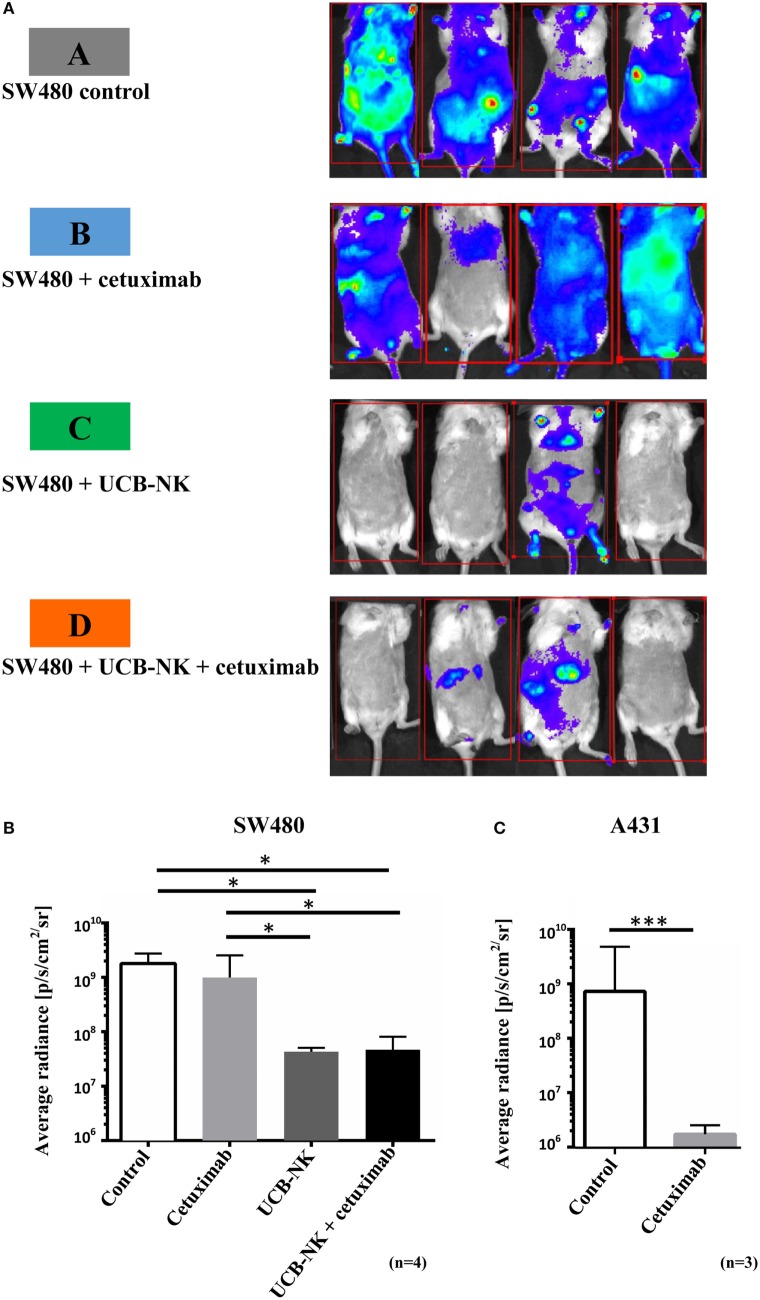
**Successful tumor elimination by UCB-NK cells as revealed by bioluminescence imaging *in vivo***. **(A)** Four mice from control and treatment groups were imaged at day 35 for tumor load and distribution. Mice were injected retro-orbitally with Gluc substrate coelenterazine and images were acquired for 5 min. In SW480 control and SW480 + cetuximab groups, tumor growth was extensive and highly disseminated, spreading to most parts of the body. However, in UCB-NK and UCB-NK + cetuximab groups, there was a significantly lower tumor load, which was further verified by calculating the average radiance between groups as shown in panel **(B)** (*n* = 4 mice per group). **(C)** Cetuximab functionality against EGFR^+++^ RAS^wt^ A431 cells was tested in parallel to SW480 studies in BRGS mice (*n* = 3 mice per group). For panels **(B,C)**, bars represent mean ± SEM. *P < 0.05 for panels **(B,C)** was calculated with unpaired *t* test.

**Figure 5:**
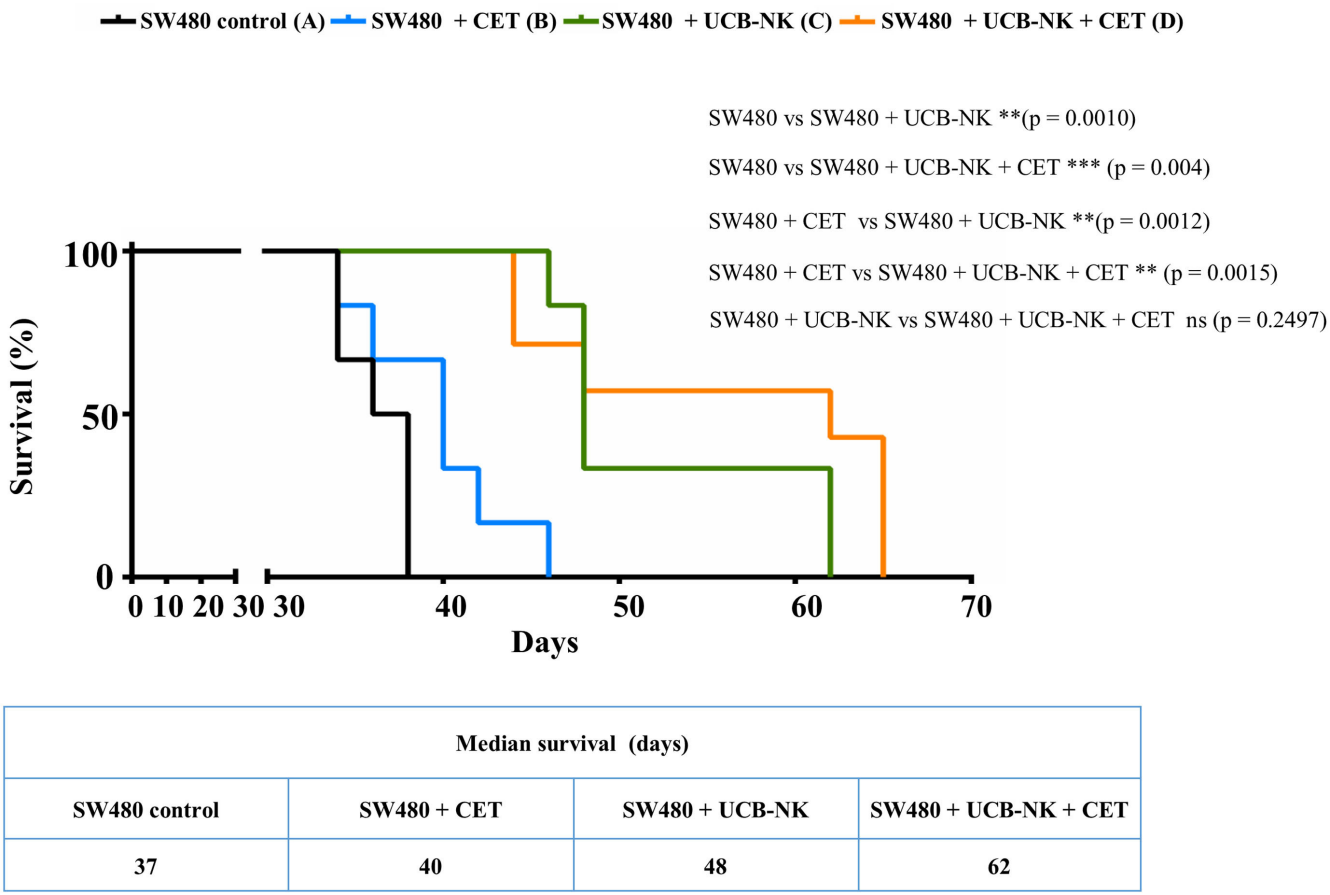
**Significant survival benefit in cetuximab-resistant RAS mutant tumor bearing mice treated with UCB-NK cells**. Kaplan–Meier survival curves were plotted for the total experimental study period from day 0 until day 65. Survival rates of SW480 (EGFR^+^, RAS^mut^) tumor-bearing mice (*n* = 6 per group) following treatment with PBS only (black line), cetuximab only (blue line), UCB-NK only (green line), and UCB-NK + cetuximab (orange line) were plotted over time to monitor treatment outcome. Statistical differences between groups were calculated using log-rank (Mantel–Cox) test and indicated in the figure.

While the blood Gluc assay measurements provided evidence of a reduction in the total tumor burden after UCB-NK treatment, we wanted to explore the impact of the therapy on the localization and size of the metastases. For that purpose, BLI was performed at day 35 after tumor inoculation. Figure 4A depicts four representative BLI images from each group at day 35 posttumor injection and average radiance from range of interest measurements are shown in Figure 4B. It is clear that mice from groups A and B showed a higher and more diffuse tumor load compared to mice treated with UCB-NK alone or in combination with cetuximab. In order to demonstrate the possibility of antitumor efficacy of cetuximab in the BRGS mouse model, we performed a similar tumor challenge using the cetuximab-sensitive A431 cell line, which bears wild-type RAS and overexpresses EGFR. A significant decrease in tumor load was observed when A431 tumors were treated with the same concentration of cetuximab as in the SW480 study (Figure 4C), confirming the *in vivo* functionality of cetuximab. We next assessed whether treatment of SW480 bearing mice with UCB-NK cells alone or in combination with cetuximab translated into a survival advantage (Figure 5). Indeed, treatment of mice with UCB-NK cells alone resulted in a significant prolongation in their life span (*P* = 0.01), whereas combinatorial therapy did not add significantly to this. Treatment with cetuximab alone did not translate into a significant survival advantage, consistent with the observed effects on tumor growth.

## Discussion

In order to test the cytotoxic potential of NK cells for treating advanced CRC patients, we compared their functional status before and after chemotherapy. We observed that peripheral blood NK cell numbers were reduced in mCRC patients and that residual NK cells were dysfunctional and unable to mount a strong effector response when stimulated with an NK cell sensitive tumor target. Though an increase in NK cell cytotoxicity was observed when tumor target cells were coated with the anti-EGFR mAb cetuximab, reflecting a capacity for ADCC, cytotoxicity was still significantly lower (both before and after chemotherapy) than that observed in healthy controls. These data indicate a decreased functional state of NK cells in patients with mCRC, which is in line with studies in mice where the cytokine production and antitumor activity of adoptively transferred NK cells were highly affected following long-term exposure to tumors (30). Through recognition of MHC class I molecules, KIRs prevent NK cells from targeting healthy cells while allowing them to detect tumor or infected cells with low or downregulated expression of MHC class I in a process known as “missing self” (31). Severely diminished or aberrant expression of MHC class I has been reported in the majority of colorectal adenocarcinomas (32, 33), which makes them an ideal target for NK cell-mediated killing. Although NK cells are infrequent in colorectal tissues (18), several independent studies investigated the clinical impact of NK cell infiltration on the prognosis of CRC, as well as in other types of carcinoma. These clinical studies, including a recent tissue microarray of 1,414 CRC biopsies, led to the conclusion that NK cell infiltration in tumors correlated with better overall response rates and progression-free survival in CRC patients (34–37), suggesting that therapies aimed at boosting NK cell functions could be beneficial in mCRC and possibly also in other types of cancer.

We evaluated and compared the cytotoxic efficacy of two different sources of feeder cell free allogeneic NK cells, i.e., A-PBNK cells and *in vitro* expanded and differentiated UCB-NK cells. *In vitro* NK cell cytotoxicity experiments revealed that the cytotoxic activity of UCB-NK cells against CRC cells was significantly higher than that of A-PBNK cells and in addition demonstrated that, while an increase in cytotoxicity through ADCC was not evident with UCB-NK cells, their cytotoxic potential was still comparable to that observed with A-PBNK potentiated by cetuximab-mediated ADCC. It is possible that the stronger cytotoxic effects of UCB-NK cells result from a more intense stimulation with cytokines in comparison to A-PBNK cells. The failure to observe ADCC-enhanced cytotoxicity with UCB-NK cells *in vitro* can be explained by their low expression levels of CD16 (29). As we previously observed *in vivo* upregulation of CD16 on UCB-NK cells upon their transfer to NOD/SCID/IL2Rgnull (NSG) mice (38), we decided to also test the efficacy of cetuximab treatment in combination with UCB-NK cells in an *in vivo* model. Treatment of SW480 RAS^mut^ tumors in BRGS mice with UCB-NK cells resulted in control of disease progression and translated into a significantly longer survival. As expected, cetuximab monotherapy did not result in a decreased SW480 tumor load or improvement in survival, recapitulating the clinical data from patients bearing RAS^mut^ CRC tumors. Unexpectedly, we failed to demonstrate superior *in vivo* antitumor effects or survival when we combined the transfer of UCB-NK cells with cetuximab infusions. The underlying causes for this latter finding remain obscure but may be related to suboptimal *in vivo* upregulation of CD16 in the used mouse model or CD16 polymorphisms in the employed batch of UCB-NK cells, both of which could have hampered efficient ADCC.

Taken together, UCB-NK cells displayed significant antitumor efficacy, suggesting a potential beneficial role for UCB-NK cells in the treatment of RAS and BRAF mutant CRC. As an important present limitation in treating mCRC patients is related to resistance to anti-EGFR mAbs, adoptive transfer of cytolytic UCB-NK cells could thus constitute a viable treatment option. Our *in vitro* and *in vivo* data demonstrating that adoptive transfer of UCB-NK cells alone was as effective as the combination of A-PBNK and cetuximab raises the possibility that UCB-NK administration could obviate the use of cetuximab in RAS^wt^ mCRC. Furthermore, UCB-NK can also lyse RAS^mut^ CRC cells at levels higher than those observed with A-PBNK. Importantly, allogeneic NK cells have demonstrated their safety in clinical trials in several solid tumors (39, 40), and more specifically, the UCB-NK cell product used in our experiments was found to be safe in a clinical trial in acute myeloid leukemia (AML) patients (Dolstra et al., 2016 manuscript submitted).

Several features make UCB-NK attractive for further clinical development. For example, our GMP-based expansion and differentiation protocol reproducibly resulted in a more than 10,000-fold expansion of cytotoxic UCB-NK cells from single donors. Furthermore, UCB-NK cells can be supplied as an “off the shelf” product, stored in large aliquots facilitating multiple infusions. Also, the low immunogenicity by UCB grafts prevents adverse reactions that are prevalent after repeated PBNK transfusions (41). In this respect, it is relevant to mention that while NK cells in general are often inhibited by recognition of MHC class I molecules on the surface of tumor cells, UCB-NK display relatively low levels of KIRs supporting their ability to effectively lyse MHC class I-expressing tumor cells (29). Finally, the ability of UCB-NK cells to proliferate and home to liver, lungs, spleen, and bone marrow after adoptive transfer has been previously demonstrated in NSG mice (38), though additional studies are required to determine whether UCB-NK cells have a similar migratory pattern upon adoptive transfer in solid tumor patients. Together, these features and observations provide UCB-NK cells with several unique advantages for further development as a universal NK cell platform.

Considering the size and heterogeneity of the tumor mass in advanced stages of CRC and other types of cancer, UCB-NK may not provide a sufficient therapeutic effect as a single agent. However, rational combinations of UCB-NK cells with existing drugs or drugs that are in clinical development can be envisioned to further increase their efficacy. Previous studies have pointed out that the proteasome inhibitor (bortezomib) (42) and the immunomodulatory drug (lenalidomide) (43) sensitize tumor cells to NK-mediated killing. In addition, UCB-NK cell application together with bispecific or trispecific antibodies that bind to tumor and UCB-NK cell-activating receptors can also increase NK cell tumor specificity (44). Though we did not specifically assess ADCC induced by other mAbs, it is very likely that the failure of UCB-NK to mediate ADCC is a more general phenomenon as this depends on binding to CD16/FcγRIII, which was found to be expressed at only low levels in the UCB-NK cell product. However, recent data from a clinical phase 1 study with the same UCB-NK cell product in patients with AML revealed significant upregulation of CD16 on UCB-NK cells post transfusion suggesting that the UCB-NK cell product may acquire the capacity to mediate ADCC in patients following adoptive transfer (Dolstra et al., manuscript submitted). Further, this phenomenon may also provide a strong rationale for combining UCB-NK cells with bispecific or trispecific killer cell engagers (45). Taken together, these approaches can substantially increase UCB-NK cell responses to advanced solid tumors, including mCRC.

In conclusion, in this study, we have demonstrated the *in vitro* efficacy of UCB-NK cells against multiple CRC cell lines independent of EGFR expression and EGFR downstream signaling mutations, and in addition have demonstrated the *in vivo* antitumor efficacy of adoptively transferred UCB-NK cells against EGFR^+^RAS^mut^ tumors. As the adoptive transfer of UCB-NK cells (oNKord^®^) has been shown to be safe in patients with AML (CCMO no. NL31699 and Dutch trial register no 2818), our data provide a rationale for the clinical exploration of UCB-NK cells in the treatment of mCRC.

## Ethics Statement

Approval for human subjects use: this study was carried out in accordance with the recommendation of the institutional review board of VU University Medical Center, Amsterdam (NCT01792934) with written informed consent from all subjects. All subjects gave written informed consent in accordance with the Declaration of Helsinki. The protocol was approved by the Committee for Scientific research of the VU University Medical Center, Cancer Center Amsterdam. Approval for animal subjects use: this study was carried out in accordance with the recommendations of the ethical committee at the Institut Pasteur (Reference # 2007–006). The protocol was approved by the French Ministry of Education and Research (Reference # 02162.01).

## Author Contributions

Conceived and designed the experiments: JV, SL-L, JPD, TG, HVV, JS, and HV. Performed the experiments: JV, SL-L, NK, and FB. Analyzed the data: JV and SL-L. Contributed reagents/materials/analysis tools: DH. Wrote the paper: JV and SL-L.

## Conflict of Interest Statement

JV, JS, NK, and FB are employees of Glycostem Therapeutics; DH serves on the scientific advisory boards of Amgen and Pfizer. JPD is a stakeholder and founder of Axenis, SAS (France). The authors declare no conflict of interest.
