# Supplementary material for: In Vivo Efficacy of Umbilical Cord Blood Stem Cell-Derived NK Cells in the Treatment of Metastatic Colorectal Cancer
Source: Front Immunol. 2017 Feb 6;8:87. doi: 10.3389/fimmu.2017.00087 (PMC5292674; doi:10.3389/fimmu.2017.00087)
Supplement: Supplementary file 1 [file data_sheet_1.pdf]

Supplementary figure 1

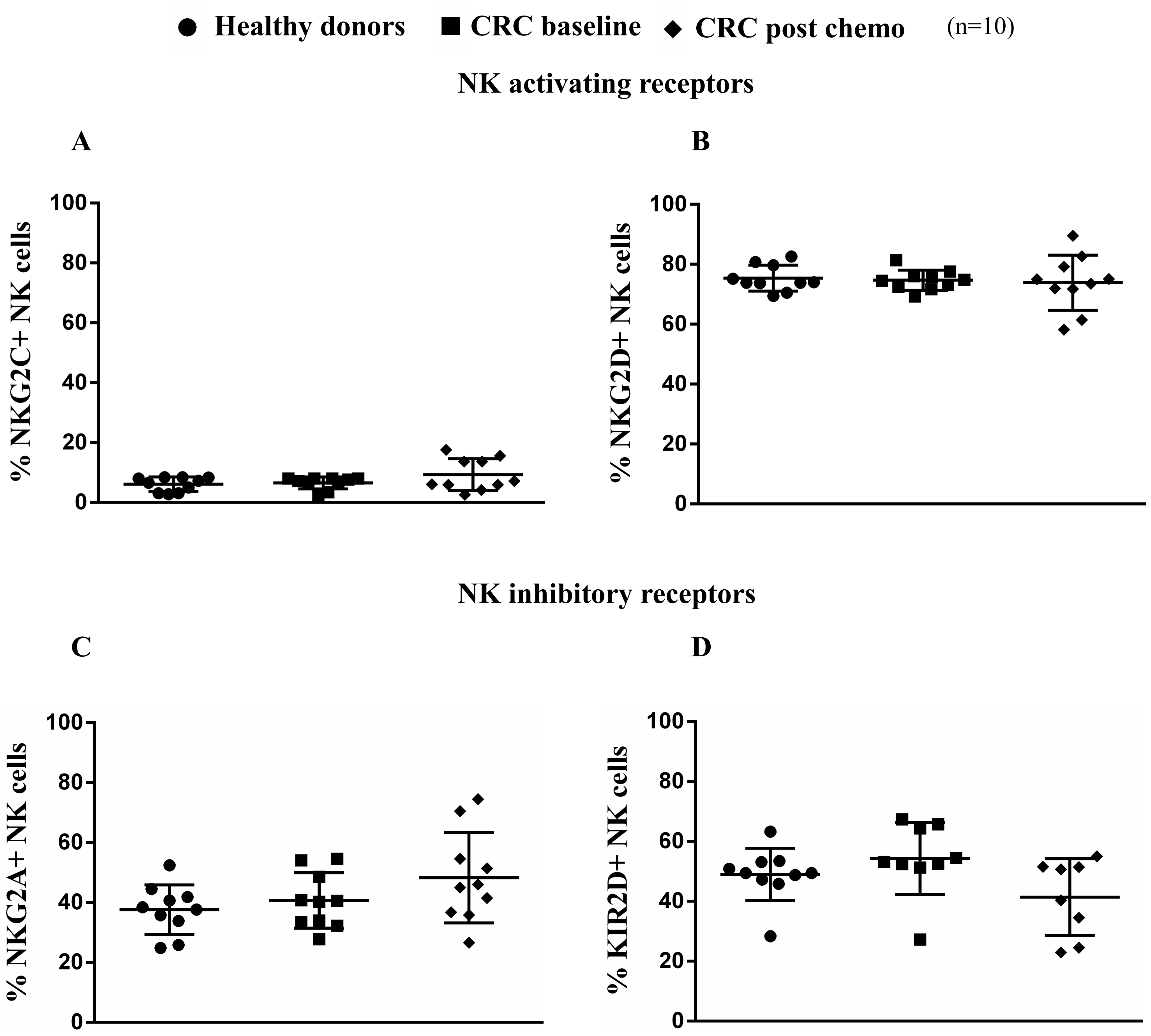

Supplementary figure 1: Expression profiles of NK cell receptors in CRC patients

Resting NK cells within PBMC populations from healthy controls and from mCRC patients at baseline and after 1 cycle of chemotherapy were monitored for the NK activating receptors NKG2D (A), NKG2C (B) and NK inhibitory receptors NKG2A (C) and KIR2D (D). Data represent mean ± SEM from 10 mCRC patients and 10 healthy controls. Statistical difference was calculated with one-way ANOVA, multiple comparison between column means.

Supplementary figure 2

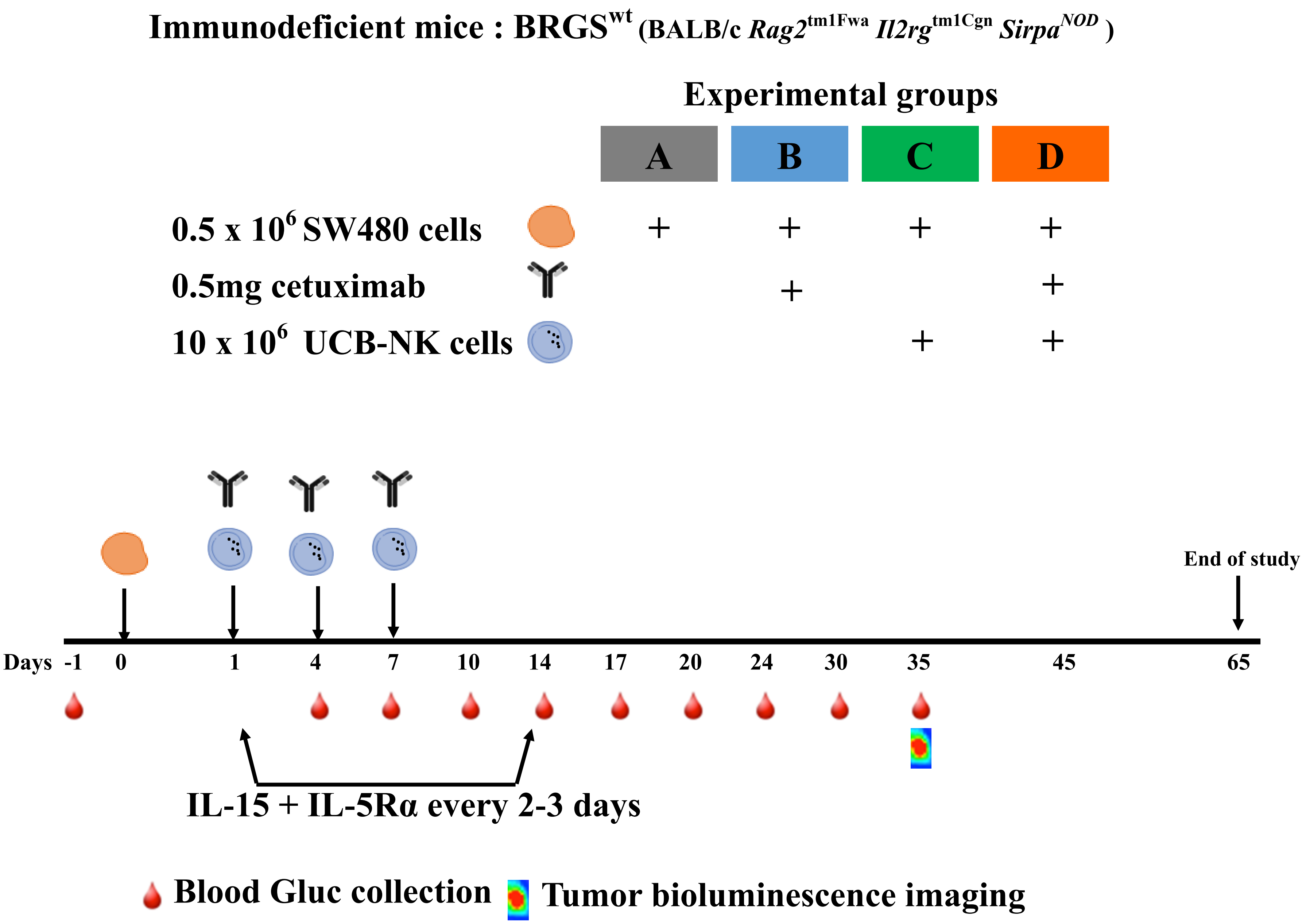

Supplementary figure 2: Schedule of *in vivo* BRGS mouse experiments.

BRGS mice were divided in 4 groups of 6 mice each. SW480 (A) is the control group, followed by treatment groups SW480 + cetuximab (B), SW480 + UCB-NK (C) and SW480 + UCB-NK + cetuximab (D). 0.5 x 10^6 Gluc transduced SW480 cells per mouse were administered i.v. to all groups at day 0. On day 1 (dose I) post tumor injection, Groups B and D mice were treated with 0.5mg cetuximab i.p. and Groups C and D were infused i.v. with 10 x 10^6 UCB-NK cells. Same doses of cetuximab and UCB-NK cells were again administered on day 4 (dose II) and day 7 (dose III) to the respective groups. 0.5µg IL-15 was mixed with 7.5 µg IL-15Rα and administered to the UCB-NK-treated groups on days 1, 4, 7, 10 and 14. Treatment effects were monitored using blood Gluc levels and BLI imaging studies, 10µl of blood was withdrawn twice a week and tumors were imaged 4 weeks after end of treatment.
